# KIR Genes and Their Ligands Predict the Response to Anti-EGFR Monoclonal Antibodies in Solid Tumors

**DOI:** 10.3389/fimmu.2016.00561

**Published:** 2016-12-05

**Authors:** Cristina Morales-Estevez, Juan De la Haba-Rodriguez, Barbara Manzanares-Martin, Ignacio Porras-Quintela, Antonio Rodriguez-Ariza, Alberto Moreno-Vega, Maria J. Ortiz-Morales, Maria A. Gomez-España, Maria T. Cano-Osuna, Javier Lopez-Gonzalez, Beatriz Chia-Delgado, Rafael Gonzalez-Fernandez, Enrique Aranda-Aguilar

**Affiliations:** ^1^Medical Oncology Department, IMIBIC, Reina Sofia University Hospital, University of Cordoba, Cordoba, Spain; ^2^Spanish Cancer Network (RTICC), Instituto de Salud Carlos III, Madrid, Spain; ^3^Immunology Department, IMIBIC, Reina Sofia University Hospital, University of Cordoba, Cordoba, Spain

**Keywords:** KIR receptor, anti-EGFR, advanced cancer, solid tumor, natural killer cells, KIR/HLA ligands

## Abstract

Killer-cell immunoglobulin-like receptors (KIRs) regulate the killing function of natural killer cells, which play an important role in the antibody-dependent cell-mediated cytotoxicity response exerted by therapeutic monoclonal antibodies (mAbs). However, it is unknown whether the extensive genetic variability of KIR genes and/or their human leukocyte antigen (HLA) ligands might influence the response to these treatments. This study aimed to explore whether the variability in KIR/HLA genes may be associated with the variable response observed to mAbs based anti-epidermal growth factor receptor (EGFR) therapies. Thirty-nine patients treated with anti-EGFR mAbs (trastuzumab for advanced breast cancer, or cetuximab for advanced colorectal or advanced head and neck cancer) were included in the study. All the patients had progressed to mAbs therapy and were grouped into two categories taking into account time to treatment failure (TTF ≤6 and ≥10 months). KIR genotyping (16 genetic variability) was performed in genomic DNA from peripheral blood by PCR sequence-specific primer technique, and HLA ligand typing was performed for HLA-B and -C loci by reverse polymerase chain reaction sequence-specific oligonucleotide methodology. Subjects carrying the KIR/HLA ligand combinations KIR2DS1/HLAC2C2-C1C2 and KIR3DS1/HLABw4w4-w4w6 showed longer TTF than non-carriers counterparts (14.76 vs. 3.73 months, *p* < 0.001 and 14.93 vs. 4.6 months, *p* = 0.005, respectively). No other significant differences were observed. Two activating KIR/HLA ligand combinations predict better response of patients to anti-EGFR therapy. These findings increase the overall knowledge on the role of specific gene variants related to responsiveness to anti-EGFR treatment in solid tumors and highlight the importance of assessing gene polymorphisms related to cancer medications.

## Introduction

The anti-human epidermal growth factor receptor (EGFR) monoclonal antibodies (mAbs) are of special interest in treating several solid metastatic tumors. Trastuzumab, cetuximab, and panitumumab plus chemotherapy prolong the survival of patients with advanced cancers (1). However, the response to treatment is not identical, due to innate differences in activity/function of an individual’s immune system, and expression of distinct genetic biomarkers that can differentially influence in the response (1, 2). Multiple hypotheses have been suggested to explain the differences in antitumor activity of these therapeutic antibodies, occurring partly to antibody-dependent cell-mediated cytotoxicity (ADCC) (3, 4), which is dependent on immune effector cells, mainly natural killer (NK) cells, binding by their Fc receptor (FcγRIII, CD16) to the Fc portion of these mAbs (5). This process conducts to the activation of the NK cells and lysis of the mAb-bound tumoral cell (6). NK cell functions are regulated by a diversity of activating and inhibitory cell surface receptors (7, 8). One of these cell surface receptors controlling the effector function of NK cells are the killer-cell immunoglobulin-like receptors (KIRs) (9). In the last decade, several KIR genes have been described; specifically, six of them are activating (KIR2DS1–5 and KIR3DS1), seven are inhibitory (KIR2DL1–3, KIR3DL1–3, and KIR2DL5), one has both properties (KIR2DL4), and two are pseudogenes (KIR2DP1 and KIR3DP1) (10–12). KIRs may either inhibit or stimulate NK cell activity after engagement with specific human leukocyte antigen (HLA) class I ligands (6, 13). HLA and KIR genes have high genetic variability (14), and KIR/HLA ligand interactions are especially diverse (15). These receptors allow the NK cells to self-discriminate healthy cells from transformed or pathogen-infected cells and regulate their effector function (16, 17).

Natural killer cells can lyse tumor cells directly by their KIR receptors. It is observed that KIR receptors specific for major histocompatibility complex (MHC) class I molecules play a major role in the anti-leukemia effect (mediating either inhibitory or activating signals) (18). Others studies have shown associations between KIR genes, their ligands, and either protection or susceptibility to solid tumors. However, the evidence for a role for KIR in solid cancer has largely been discussed (19, 20). It has been also suggested that NK cells in combination with mAbs may confer more rapid killing of tumor cells, due to the additive benefit of the two modalities of treatment and the potent cytotoxic capability of NK cells (21). Currently, it is unknown whether these KIR receptors might influence the response to treatment with mAbs in solid cancer. Because of the extensive genetic variability of KIR and/or their HLA ligands and the importance of these combinations in the response of NK cells, the present study aimed to explore whether the variability in KIR/HLA genes may be associated with the variable response observed to mAbs based anti-EGFR therapies.

## Materials and Methods

The study was designed and performed by the authors, and the protocol and all amendments were presented and approved by the Ethics and Research Committee of Hospital Universitario Reina Sofia, Cordoba, Spain, local ethics committees, all of which follow the Helsinki Declaration and good clinical practices (March 25, 2013). The experimental protocol conforms to International Ethical Standards.

### Calculation of Sample Size

The number of patients to include in the analysis to find differences of at least 30% between the groups time to treatment failure (TTF) ≤6 and ≥10 months considering a minimum frequency of 30% for the KIR polymorphisms was 33 patients with a confidence level of 95% and a statistical power of 80%.

### Population

The patients included in this study (*n* = 39) were eligible taking into account the next inclusion criteria: (A) aged 18 years or older; (B) signed informed consent; and (C) all patients should have been treated with anti-EGFR therapy (trastuzumab for advanced breast cancer and cetuximab for head and neck cancer or advanced colorectal). All the patients had progressed before 6 months treatment with anti-EGFR mAb (short TTF, TTF ≤ 6 months, *n* = 19) or after 10 months (long TTF, TTF ≥ 10 months, *n* = 20), establishing two extreme phenotypes of response (TTF ≤6 and ≥10 months). These two groups of analysis are established, taking into account the median time to progression published in control and treatment arms in the pivotal studies (22, 23).

### KIR and HLA Genotyping

Genomic DNA was extracted from peripheral blood samples drawn in ethylenediaminetetraacetic acid (EDTA) anticoagulant tubes using Maxwell 16 Instrument to provide an automated method of purification of nucleic acids (Promega Corporation, Madison, WI, USA) and was typed for both KIR and HLA class I alleles following the manufacturer’s instructions. HLA-A, -B, and -C genotyping were performed with the INNO-LIPA HLA-A Multiplex, HLA-B Multiplex plus, and HLA-C kits, respectively (Fujirebio Europe N.V., Gante, Belgium), using HLA-specific primers for nucleic acid amplification of the different Loci. HLA-Bw4 primers for exon 2 of the HLA-Bw4 alleles were also used. These are based on the polymerase chain reaction sequence-specific oligonucleotide (PCR-SSO) reverse method. Then, HLA alleles were determined using the LIRASTM software for INNO-LIPA HLA. SSP technique was used on samples that have failed to be analyzed by SSO or for situations where higher resolutions were required. These analyses were made using A, B, and C locus High Res SSP Unitray Kits (Invitrogen by Life Technologies Corporation, Brown Dee, WI, USA). KIR genotyping was performed using sequence-specific primers (KIR Ready gene of Inno-train Diagnostik GmbH, Kronberg, Taunus, Germany) able to detect the presence of 14 different KIR genes (2DL1, 2DL2, 2DL3, 2DL4, 2DL5, 2DS1, 2DS2, 2DS3, 2DS4, 2DS5, 3DL1, 3DL2, 3DL3, and 3DS1), 2 pseudogenes (2DP1 and 3DP1), and the common variants of KIRDL5, the KIR2DS4 allele, and KIR3DP1 allele. This method provided a high degree of resolution, since each primer pair identifies two linked, *cis-*located polymorphic sites. Genotyping was done at a level of resolution, which allowed determining the KIR-binding epitope to distinguish Bw4 specificities and the HLA-C dimorphism at position 80 of the α1 helix. HLA alleles were grouped into four major categories based on the amino acid sequence determining the KIR-binding epitope in HLA-C and HLA-B molecules. HLA-C alleles are of the C1 or C2 group, and HLA-B alleles can be classified as either Bw4 or Bw6. HLA-C allotypes express C1 epitopes (characterized by an asparagine in position 80) or C2 epitopes (sharing a lysine in position 80). Moreover, two HLA-B alleles (HLA-B*46:01 and B*73:01) were considered as C1 group (24). All HLA-B molecules, and some HLA-A molecules, can be classified as either Bw4 or Bw6 allotypes on the basis of residues at positions 77–83 in the A1 domain. Among Bw4^+^ HLA-A allotypes, HLA-A*24:02, A*32:01, and A*23:01, but not HLA-A*25:01, were considered (25). However, HLA-A*03 and *11 ligands for KIR3DL2 were not studied.

### Statistical Methods

Fisher’s exact and Chi-square tests were used to examine the association between two categorical variables [i.e., KIRs/HLA pairs vs. duration of the anti-EGFR therapy (≤6 or ≥10 months)]. For time variables (duration of therapy), the Kaplan–Meier and log-rang tests were used. Statistical significance was defined as a two-tailed *p*-value <0.05, and a *p*-value from 0.05 to 0.1 was regarded as marginally statistically significant. Due to the exploratory nature of this study, no multiplicity adjustment was made for significance tests, using the SPSS program.

## Results

The characteristics of the included patients are shown in Table 1. Patients were grouped into two categories according to the progression criteria to the anti-EGFR therapy: short TTF (≤6 months, *n* = 20) or long TTF (≥10 months, *n* = 19) (22, 23). All breast cancer over expressed HER2, and all colorectal cancers were RAS wild type.

**Table 1:** **Characteristics of patients according to the time to treatment failure (TTF ≤6 or ≥10 months)**.

		TTF	
		≤6 months	≥10 months
Gender	Female	10 (43.48%)	13 (56.52%)
	Male	10 (62.5%)	6 (37.5%)

Primary tumor	Breast	5 (33.33%)	10 (66.67%)
	Colon	10 (62.5%)	6 (37.5%)
	Head and neck	5 (62.5%)	3 (37.5%)

Primary tumor diagnosis age (median)		59	59

Metastatic disease diagnosis age (median)		59	59

Grade	2	13 (61.9%)	8 (38.1%)
	3	5 (50%)	5 (50%)
	Unknown	2 (25%)	6 (75%)

Breast cancer ER	+	5 (55.56%)	4 (44.44%)
	−	0 (0%)	6 (100%)
	
PR	+	5 (55.56%)	4 (44.44%)
	−	0 (0%)	6 (100%)
	
HER2	Overexpressed	5 (33.33%)	10 (66.67%)
	Not overexpressed	0 (0%)	0 (0%)

Colon cancer	WT	10 (62.5%)	6 (37.5%)
RAS	Mutated	0 (0%)	0 (0%)

*ER, estrogen receptor; PR, progesterone receptor; RAS, RAS mutation status*.

### KIR Genotypes and HLA Ligand Polymorphisms

Table 2 lists all the inhibitory and activating KIR genes, and pseudogenes evaluated in this study; after analyzing the frequencies in the two groups, non-significant differences were observed in the inhibitory, activating, and pseudogenes KIR genes.

**Table 2:** **Lists all the inhibitory, activating KIR genes, and pseudogenes according to the time to treatment failure (TTF ≤6 or ≥10 months)**.

Inhibitory KIR genes presence		TTF < 6 months	TTF > 10 months
2DL1	Yes	18 (50%)	18 (50%)
	No	2 (66.7%)	1 (33.3%)
2DL2	Yes	12 (48%)	13 (52%)
	No	8 (57.1%)	6 (42.9%)
2DL3	Yes	18 (51.4%)	17 (48.6%)
	No	2 (50%)	2 (50%)
2DL5	Yes	10 (43.5%)	13 (56.5)
	No	10 (62.5%)	6 (37.5%)
3DL1	Yes	19 (54.3%)	16 (45.7%)
	No	1 (25%)	3 (75%)
3DL2^a^	Yes	20 (51.3%)	19 (48.7%)
	No	0	0
3DL3^a^	Yes	20 (51.3%)	19 (48.7%)
	No	0	0
2DL4^a^	Yes	20 (51.3%)	19 (48.7%)
	No	0	0

**Activating KIR genes presence**		**TTF < 6 months**	**TTF > 10 months**

2DS1	Yes	9 (37.5%)	15 (62.5%)
	No	11 (73.3%)	4 (26.7%)
2DS2	Yes	12 (48%)	13 (52%)
	No	8 (57.1%)	6 (42.9%)
2DS3	Yes	3 (27.3%)	8 (72.7%)
	No	17 (60.7%)	11 (39.3%)
2DS4	Yes	19 (52.8%)	17 (47.2%)
	No	1 (33.3%)	2 (66.7%)
2DS5	Yes	9 (47.4%)	10 (52.6%)
	No	11 (55%)	9 (45%)
3DS1	Yes	9 (45%)	11 (55%)
	No	11 (57.9%)	8 (42.1%)
2DL4^a^	Yes	20 (51.3%)	19 (48.7%)
	No	0	0

**KIR pseudogene presence**		**TTF < 6 months**	**TTF > 10 months**

2DP1	Yes	18 (51.4%)	17 (48.6%)
	No	2 (50%)	2 (50%)
3DP1^a^	Yes	20 (51.3%)	19 (48.7%)
	No	0	0

*^a^Present in 100% of the population*.

According to the expected KIR framework genes, KIR2DL4, KIR3DL2, KIR3DL3, and KIR3DP1 were present in all patients among groups, suggesting the correct internal controls. Table 3 shows their corresponding HLA ligands (HLA-C1, HLA-C2, HLABw4, or HLABw6) and the frequency distributions of taking into account the TTF (≤6 or ≥10 months).

**Table 3:** **HLA according to the time to treatment failure (TTF ≤6 or ≥10 months)**.

HLA		TTF ≤ 6 months	TTF ≥ 10 months
HLA-B	w4w4	3 (33.3%)	6 (66.7%)
	w4w6	13 (52%)	12 (48%)
	w6w6	4 (80%)	1 (20%)
HLA-C	C1C1	7 (77.8%)	2 (22.2%)
	C1C2	8 (38%)	13 (62%)
	C2C2	5 (55.5%)	4 (44.5%)

The frequencies of the HLA class I ligands of the KIR (Bw4, C1, and C2 in homozygosity and heterozygosity) were analyzed. As previously described, different groups (Bw4/Bw4, Bw4/Bw6, or Bw6/Bw6 for HLA-B and C1/C1, C1/C2, and C2C2 for HLA-C) were defined on the basis to homozygosis or heterozygosis status. We observed non-significant differences in the frequency between two groups (TTF ≤6 or ≥10 months).

### KIR–HLA Ligand Combinations

**Figure 1:**
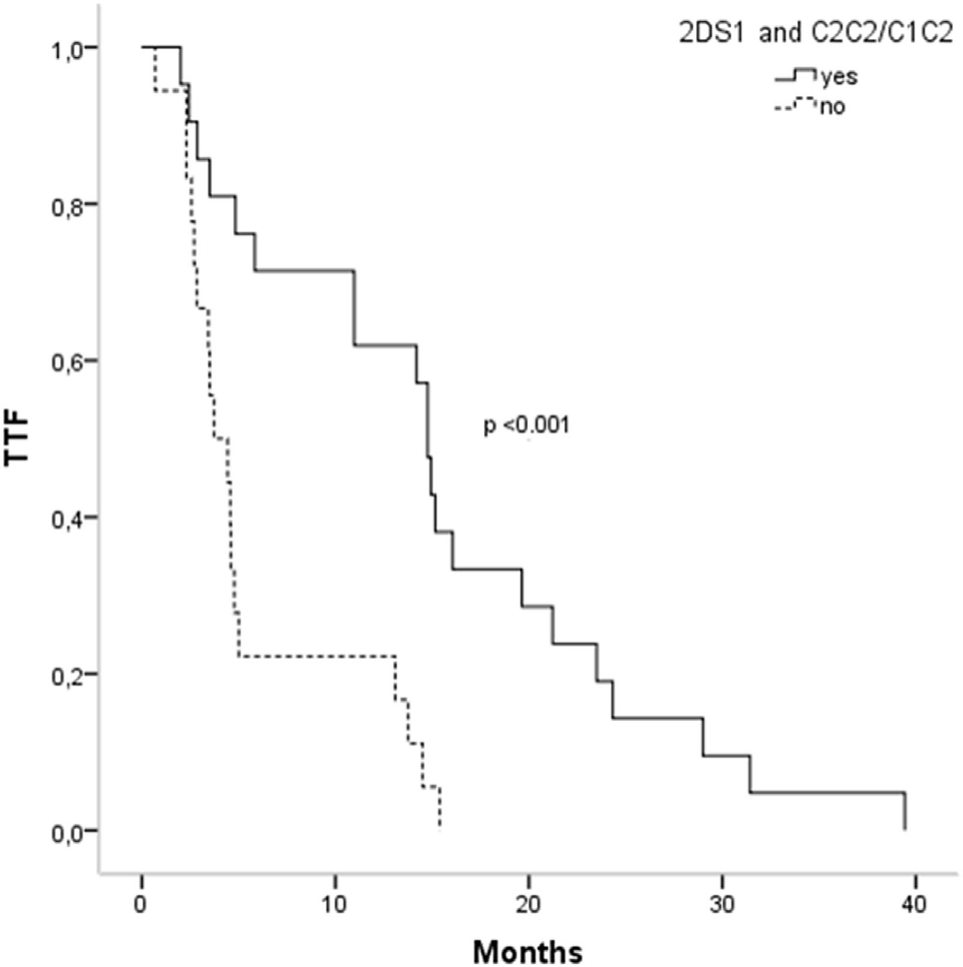
**Time to treatment failure (TTF) according to 2DS1 polymorphisms and their ligands**.

**Figure 2:**
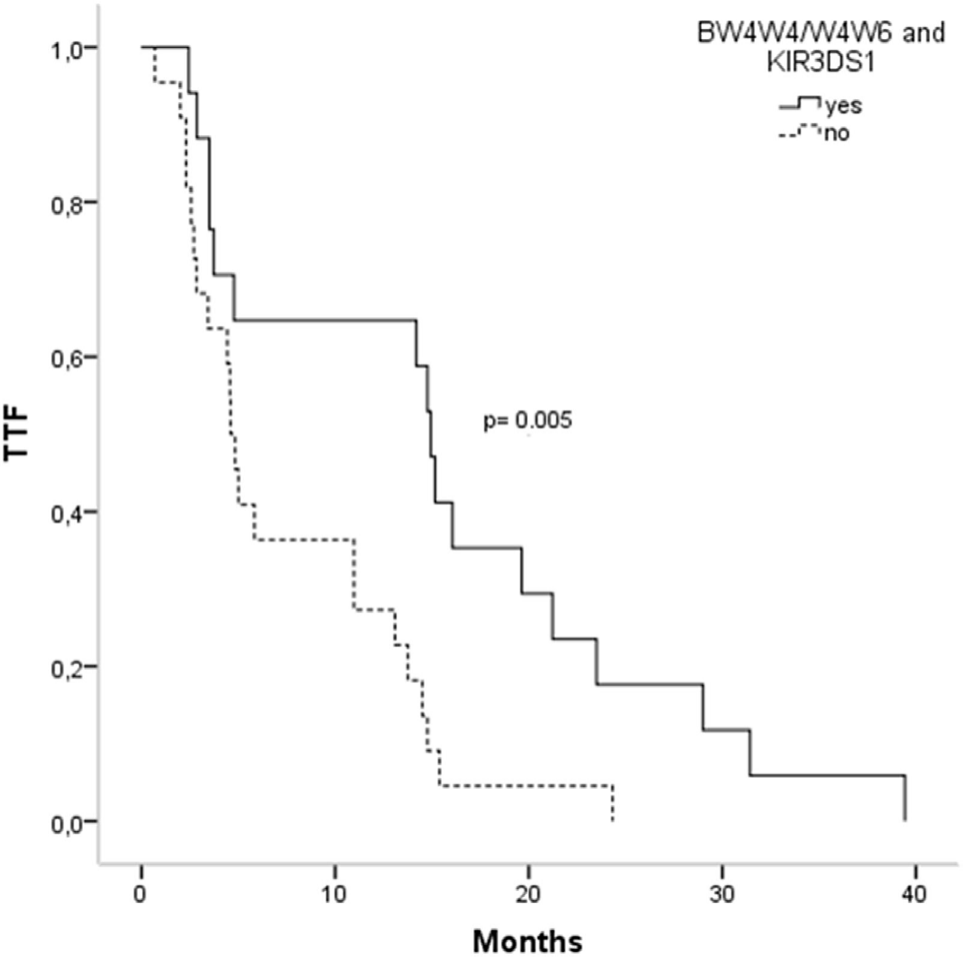
**Time to treatment failure (TTF) according to 3DS1 polymorphisms and their ligands**.

Activating and inhibitory combinations with different KIR genotypes and their HLA ligands are shown in Table 4. TTF, according to the KIR–HLA ligand combinations, was explored. We found a significant association between the activating combinations KIR2DS1/HLAC2C2-C1C2 with the TTF > 10 months (*p* = 0.002) and a statistical trend within KIR3DS1/HLABw4w4-w4w6 with the TTF > 10 months (*p* = 0.079). Interestingly, subjects carrying these two activating combinations showed longer TTF than non-carriers; KIR2DS1/HLAC2C2-C1C2, TTF: 14.76 vs. 3.73 months (*p* < 0.001) (Figure 1), and KIR3DS1/HLABw4w4-w4w6, TTF: 14.93 vs. 4.6 months (*p* = 0.005) (Figure 2). With 2DS1, the difference was observed only when the ligand was C1C2; similarly, the same occurs with 3DS1with their ligands (differ only for heterozygotes).

**Table 4:** **Activating and inhibitory combinations with different KIR genotypes and their HLA ligands according to the time to treatment failure (TTF ≤6 or ≥10 months)**.

KIR/HLA activating combinations presence	TTF ≤ 6 months	TTF ≥ 10 months	*p*
KIR 2DS1-HLA C2C2 or C1C2			
Yes	6 (28.6%)	15 (71.4%)	*p* = 0.002
No	14 (77.8%)	4 (22.2%)	
KIR 2DS2-HLA C1C1 or C1C2			
Yes	9 (45%)	11 (55%)	
No	11 (57.9%)	8 (42.1%)	*p* = 0.42
KIR 3DS1-HLA w4w4 or w4w6			
Yes	6 (35.3%)	11 (64.7%)	*p* = 0.079
No	14 (63.6%)	8 (36.4%)	

**KIR/HLA inhibitory combinations presence**	**TTF ≤ 6 months**	**TTF ≥ 10 months**	***p***

2DL1-HLA C2C2 or C1C2			
Yes	14 (48.3%)	15 (51.7%)	*p* = 0.52
No	6 (60%)	4 (40%)	
2DL2-HLA C1C1 or C1C2			
Yes	9 (45%)	11 (55%)	*p* = 0.42
No	11 (57.9%)	8 (42.1%)	
2DL3-HLA C1C1 or C1C2			
Yes	14 (50%)	14 (50%)	*p* = 0.79
No	6 (54.5%)	5 (45.5%)	
3DL1-HLA w4w4 or w4w6			
Yes	16 (51.6%)	15 (48.4%)	*p* = 0.93
No	4 (50%)	4 (50%)	

## Discussion

Our results provide new evidence indicating that two activating combinations of KIRs and their HLA ligands predict longer TTF in patients treated with mAbs based anti-EGFR therapies. Specifically, subjects carrying the KIR2DS1/HLAC2C2-C1C2 and KIR3DS1/HLABw4w4-w4w6 showed longer TTF than non-carriers. These findings increase the knowledge on the role of specific variants of KIRs related to responsiveness to anti-EGFR treatment in solid tumors.

Understanding of the variety of KIR/HLA ligands interactions is improving, but our findings and others studies are revealing increasing levels of complexity. KIR/HLA class I interaction is a clear example of genetic epistasis in which the presence of receptor/ligand pairs is necessary for the induction of functional activity, while the presence of one but the absence of the other is not sufficient to influence NK cell function. It is becoming clear, however, that the interactions between the extensive polymorphism KIR and their HLA ligands can alter immune function to influence susceptibility to several diseases including hematological and solid cancer. Most of these studies only explored the association between the presence of KIR/HLA frequencies in cancer with the aim to test the impact of KIR/HLA status on individual’s susceptibility (26). A recent study performed in patients with metastatic colon cancer demonstrated that the genotyping KIR/HLA pairs helps to predict overall survival to treatment with FOLFIRI (27). In the same context, the critical early role of NK cells in facilitating response to imatinib in patients with chronic phase-chronic myeloid leukemia (CP-CML) that cannot be overcome by subsequent intensification of therapy is well known. KIR genotyping may add valuable prognostic information to future baseline predictive scoring systems in CP-CML patients and facilitate optimal frontline treatment selection (28). Patients with non-small lung cancer, positive for KIR2DL2 and KIR2DS2 gene, and homozygous for the C1 ligand were six times more likely to respond to treatment than those with other genotypes. In accordance with this, patients with the KIR2DL2+/KIR2DS2+, C1C1 genotype survived longer than others (29).

It is well known that the activity of NK cells is controlled by a balance of signals generated from the cell surface receptor inhibitors and activators. Furthermore, some of the clinically approved therapeutic mAbs to treat solid tumor are considered to function partially through triggering NK cell-mediated ADCC activity (30). A recent study suggests that transfer of allogeneic NK cells in combination with a cancer-targeting antibody such as trastuzumab may represent an effective approach to adoptive immunotherapy (31). In this regard, it was found that ADCC mediated by allogeneic NK cells occurred despite combinations of NK cells and breast cancer targets predicted to trigger inhibitory KIR signaling. Overall, in spite of the central role of NK cells in host immune responses and the fact that the KIR/HLA gene system is the main receptor system able to modulate NK cell function, it is unknown whether these KIR/HLA ligands combinations could influence the response to anti-EGFR therapies. It would be possible that the efficiency of anti-EGFR antibody is stronger due to the target cells are lysed, on one hand, through ADCC and, on the other, through the lack of inhibitory signal or even through an activating signal due to KIR2DS1 and the binding of HLA-I corresponding allele. In addition, some groups have studied the relationship between different genotypes gamma receptor Fc and effectiveness of therapy anti EGFR with conflicting results (32). A reason for that could be explained due to the participation of other important variables in ADCC, such as KIR different genotypes.

In addition to NK cells having inhibitory KIR receptors with a lower avidity to HLA-ligand (thus, having a decreased inhibitory function respect to other KIRs) and having several activating KIR receptors may induce an increase NK-mediated cytolysis of target cells. Therefore, our results support the hypothesis that the presence of activating KIRs (KIR2DS1/C2 and KIR3DS1/HLA-Bw4) favors longer TTF after anti-EGFR treatment.

However, no relationships between the presence/absence of KIR inhibitors and TTF were evident. At the cellular level, these effects could be mediated by an increase in activating KIR on NK cells and HLA ligands specific in tumor cells, which in turn may improve tumor NK-mediated immunity. However, the controversy of what is the reason NK cells, which under normal conditions not by activating receptors are activated to prevent autoimmune diseases, are released at this time arises to exercise its function. Another proposed mechanism is related to the presentation by HLA of “*de novo*.” mAb treatment could modify the complexes peptide/HLA influencing the function of NK cells in the tumoral microenvironment due to the increasing of activators KIR function (33). Similarly, it is possible that some peptides (new or only more abundant) may occur by HLA to KIR during tumor growth or even due to the treatment with mAb. In our case, it would be due to a predominance of KIR/activating ligand (2DS1/C2) on its corresponding inhibitory combination KIR/HLA (2DL1/C2). These activating KIRs would activate the sensitive NK cells or by the appearance of the previously mentioned new peptides (the possibility that in addition to HLA, KIR activators have other ligands present in healthy cells and would be present in these circumstances) or because activation by mAbs of a potent NK cell response; it is well known that when NK is very active, their activator KIR is functional (34).

The other mechanism for this hyperactivated microenvironment is the local secretion of citoquinas IL-12 and IL-15. When NK cells are activated *via* IL-12 and IL-15, this protective mechanism is released and lyses tumor cells by 2DS1 (35). The activation may be initiated due to the treatment with mAbs (trastuzumab or cetuximab) by NK CD16 pathway and this activation release IFN gamma other cytokines that may activate macrophages, which release more mediators increasing NK activity.

Another issue that supports the results observed in our study is the fact that 2DS1 is associated with homozygosity or heterozygosity C2. *In vitro* studies showed that NK cells from donors with 2DS1 C2C2 were not able to lyse C2-presenting targets cells. Thus, C2C2 subjects would be hardly able to activate NK *via* 2DS1, and their activation would occur only in C1C2 subjects. In agreement, this study found that 2DS1/C1C2 subjects had a longer TTF (data not shown). Under normal condition, these activating receptors are inhibited in NK cells to prevent autoimmune response. However, there is controversy on this mechanism on activation under pathological conditions, including tumoral progression. In addition to the possible predictive value of KIRs receptors for the response to treatment with mAbs, our results support the potential therapeutic value of pharmacological modulation of KIR activity.

The current study has several limitations. First, the study sample includes a cohort of patients suffering from different tumors that have been pooled together, although all of them are under anti-EGFR therapy and are advanced solid tumors. Therefore, we cannot generalize our results to other kind of cancer or therapy. Another point is that our findings should be interpreted within the context of the experimental limitations, so the causal nature of the relationship between the interaction of KIR and HLA-I ligands and the delay in TTF remains uncertain and the potential mechanisms should be explored and validated in future studies.

## Conclusion

Our results showed that two activating KIR/HLA ligand combinations predict better response of patients to anti-EGFR therapy. Future studies, currently underway, should confirm these results and support the possible predictive and therapeutic value of different KIR genotypes and its pharmacological modulation, in combination with mAbs in the treatment of solid tumors.

## Author Contributions

Full access to the data in the study and responsibility for the integrity of the data and the accuracy of the data analysis: CM-E, EA-A, and JH-R. Conception and design of the study: CM-E and JH-R. Provision of study materials or subjects: CM-E, JH-R, IP-Q, AM-V, MO-M, MG-E, MC-O, JL-G, and BC-D. Collection and assembly of data: CM-E, RG-F, and JH-R. Analysis and interpretation: RG-F and BM-M. Drafting of the manuscript: CM-E, RG-F, and JH-R. Critical review for important intellectual content: EA-A, AR-A, and JH-R. All the authors read and approved the final manuscript.

## Conflict of Interest Statement

The authors declare that the research was conducted in the absence of any commercial or financial relationships that could be construed as a potential conflict of interest.

## Abbreviations

ADCC, antibody-dependent cell-mediated cytotoxicity; EDTA, ethylenediaminetetraacetic acid; EGFR, epidermal growth factor receptor; KIRs, killer-cell immunoglobulin-like receptors; mAbs, monoclonal antibodies; NK, natural killer; PCR-SSO, polymerase chain reaction sequence-specific oligonucleotide; TTF, time to treatment failure.

## References

[1] Hall PS Cameron DA. Current perspective – trastuzumab. Eur J Cancer (2009) 45:12–8. 10.1016/j.ejca.2008.10.013 19042123

[2] Rivero-Juarez A Gonzalez R Frias M Manzanares-Martin B Rodriguez-Cano D Perez-Camacho I KIR2DS2 as predictor of thrombocytopenia secondary to pegylated interferon-alpha therapy. Pharmacogenomics J (2016). 10.1038/tpj.2016.19 26975229

[3] Varchetta S Gibelli N Oliviero B Nardini E Gennari R Gatti G Elements related to heterogeneity of antibody-dependent cell cytotoxicity in patients under trastuzumab therapy for primary operable breast cancer overexpressing Her2. Cancer Res (2007) 67:11991–9. 10.1158/0008-5472.CAN-07-2068 18089830

[4] Menard S Pupa SM Campiglio M Balsari A Fagnoni F Costa A Apoptosis induction by trastuzumab: possible role of the core biopsy intervention. J Clin Oncol (2005) 23:7238–40. 10.1200/JCO.2005.02.4679 16192615

[5] Clynes RA Towers TL Presta LG Ravetch JV. Inhibitory Fc receptors modulate in vivo cytotoxicity against tumor targets. Nat Med (2000) 6:443–6. 10.1038/74704 10742152

[6] Caligiuri MA. Human natural killer cells. Blood (2008) 112:461–9. 10.1182/blood-2007-09-077438112/3/461 18650461 PMC2481557

[7] Bakker AB Wu J Phillips JH Lanier LL. NK cell activation: distinct stimulatory pathways counterbalancing inhibitory signals. Hum Immunol (2000) 61:18–27. 10.1016/S0198-8859(99)00160-3 10658974

[8] Valiante NM Uhrberg M Shilling HG Lienert-Weidenbach K Arnett KL D’Andrea A Functionally and structurally distinct NK cell receptor repertoires in the peripheral blood of two human donors. Immunity (1997) 7:739–51. 10.1016/S1074-7613(00)80393-3 9430220

[9] Vilches C Parham P. KIR: diverse, rapidly evolving receptors of innate and adaptive immunity. Annu Rev Immunol (2002) 20:217–51. 10.1146/annurev.immunol.20.092501.134942092501.134942 11861603

[10] Williams AP Bateman AR Khakoo SI. Hanging in the balance. KIR and their role in disease. Mol Interv (2005) 5:226–40. 10.1124/mi.5.4.6 16123537

[11] Barbour JD Sriram U Caillier SJ Levy JA Hecht FM Oksenberg JR Synergy or independence? Deciphering the interaction of HLA class I and NK cell KIR alleles in early HIV-1 disease progression. PLoS Pathog (2007) 3:e43 10.1371/journal.ppat.0030043 17447840 PMC1853116

[12] Uhrberg M. The KIR gene family: life in the fast lane of evolution. Eur J Immunol (2005) 35:10–5. 10.1002/eji.200425743 15580655

[13] Faure M Long EO. KIR2DL4 (CD158d), an NK cell-activating receptor with inhibitory potential. J Immunol (2002) 168:6208–14. 10.4049/jimmunol.168.12.6208 12055234

[14] Kulkarni S Martin MP Carrington M. The Yin and Yang of HLA and KIR in human disease. Semin Immunol (2008) 20:343–52. 10.1016/j.smim.2008.06.003 18635379 PMC3501819

[15] Falco M Moretta L Moretta A Bottino C. KIR and KIR ligand polymorphism: a new area for clinical applications? Tissue Antigens (2013) 82:363–73. 10.1111/tan.12262 24498992

[16] Nutt SL Brady J Hayakawa Y Smyth MJ. Interleukin 21: a key player in lymphocyte maturation. Crit Rev Immunol (2004) 24:239–50. 10.1615/CritRevImmunol.v24.i4.20 15588224

[17] Rivero-Juarez A Gonzalez R Camacho A Manzanares-Martin B Caruz A Martinez-Peinado A Natural killer KIR3DS1 is closely associated with HCV viral clearance and sustained virological response in HIV/HCV patients. PLoS One (2013) 8:e61992. 10.1371/journal.pone.0061992 23613999 PMC3629002

[18] Moretta A Pende D Locatelli F Moretta L. Activating and inhibitory killer immunoglobulin-like receptors (KIR) in haploidentical haemopoietic stem cell transplantation to cure high-risk leukaemias. Clin Exp Immunol (2009) 157:325–31. 10.1111/j.1365-2249.2009.03983.x 19664139 PMC2745025

[19] Ivarsson MA Michaelsson J Fauriat C. Activating killer cell Ig-like receptors in health and disease. Front Immunol (2014) 5:184. 10.3389/fimmu.2014.00184 24795726 PMC4001058

[20] Ozturk OG Gun FD Polat G. Killer cell immunoglobulin-like receptor genes in patients with breast cancer. Med Oncol (2012) 29:511–5. 10.1007/s12032-011-9932-x 21479698

[21] Stein MN Shin J Gudzowaty O Bernstein AM Liu JM. Antibody-dependent cell cytotoxicity to breast cancer targets despite inhibitory KIR signaling. Anticancer Res (2006) 26:1759–63. 16827104

[22] Slamon DJ Leyland-Jones B Shak S Fuchs H Paton V Bajamonde A Use of chemotherapy plus a monoclonal antibody against HER2 for metastatic breast cancer that overexpresses HER2. N Engl J Med (2001) 344:783–92. 10.1056/NEJM200103153441101 11248153

[23] Van Cutsem E Kohne CH Hitre E Zaluski J Chang Chien CR Makhson A Cetuximab and chemotherapy as initial treatment for metastatic colorectal cancer. N Engl J Med (2009) 360:1408–17. 10.1056/NEJMoa0805019 19339720

[24] Moesta AK Norman PJ Yawata M Yawata N Gleimer M Parham P. Synergistic polymorphism at two positions distal to the ligand-binding site makes KIR2DL2 a stronger receptor for HLA-C than KIR2DL3. J Immunol (2008) 180:3969–79. 10.4049/jimmunol.180.6.3969 18322206

[25] Stern M Ruggeri L Capanni M Mancusi A Velardi A. Human leukocyte antigens A23, A24, and A32 but not A25 are ligands for KIR3DL1. Blood (2008) 112:708–10. 10.1182/blood-2008-02-137521blood-2008-02-137521 18502829

[26] Jobim MR Jobim M Salim PH Portela P Jobim LF Leistner-Segal S Analysis of KIR gene frequencies and HLA class I genotypes in breast cancer and control group. Hum Immunol (2013) 74:1130–3. 10.1016/j.humimm.2013.06.021 23792055

[27] De Re V Caggiari L De Zorzi M Talamini R Racanelli V D’Andrea M Genetic diversity of the KIR/HLA system and outcome of patients with metastatic colorectal cancer treated with chemotherapy. PLoS One (2014) 9:e84940. 10.1371/journal.pone.0084940 24497922 PMC3908861

[28] Yeung DT Tang C Vidovic L White DL Branford S Hughes TP KIR2DL5B genotype predicts outcomes in CML patients treated with response-directed sequential imatinib/nilotinib strategy. Blood (2015) 126:2720–3. 10.1182/blood-2015-07-655589 26500342

[29] Wisniewski A Jankowska R Passowicz-Muszynska E Wisniewska E Majorczyk E Nowak I KIR2DL2/S2 and HLA-C C1C1 genotype is associated with better response to treatment and prolonged survival of patients with non-small cell lung cancer in a Polish Caucasian population. Hum Immunol (2012) 73:927–31. 10.1016/j.humimm.2012.07.323 22836042

[30] Wang W Erbe AK Hank JA Morris ZS Sondel PM. NK cell-mediated antibody-dependent cellular cytotoxicity in cancer immunotherapy. Front Immunol (2015) 6:368. 10.3389/fimmu.2015.00368 26284063 PMC4515552

[31] Dahlberg CI Sarhan D Chrobok M Duru AD Alici E. Natural killer cell-based therapies targeting cancer: possible strategies to gain and sustain anti-tumor activity. Front Immunol (2015) 6:605. 10.3389/fimmu.2015.00605 26648934 PMC4663254

[32] Calemma R Ottaiano A Trotta AM Nasti G Romano C Napolitano M Fc gamma receptor IIIa polymorphisms in advanced colorectal cancer patients correlated with response to anti-EGFR antibodies and clinical outcome. J Transl Med (2012) 10:232. 10.1186/1479-5876-10-232 23171437 PMC3551834

[33] Cassidy SA Cheent KS Khakoo SI. Effects of peptide on NK cell-mediated MHC I recognition. Front Immunol (2014) 5:133. 10.3389/fimmu.2014.00133 24744756 PMC3978238

[34] Long EO Kim HS Liu D Peterson ME Rajagopalan S. Controlling natural killer cell responses: integration of signals for activation and inhibition. Annu Rev Immunol (2013) 31:227–58. 10.1146/annurev-immunol-020711-075005 23516982 PMC3868343

[35] Foley B Felices M Cichocki F Cooley S Verneris MR Miller J. The biology of NK cells and their receptors affects clinical outcomes after hematopoietic cell transplantation (HCT). Immunol Rev (2014) 258:45–63. 10.1111/imr.12157 24517425 PMC3927144

